# Early changes in coagulation but not inflammatory biomarkers under intermittent ART: the randomized ANRS 106 WINDOW trial

**DOI:** 10.7448/IAS.17.4.19551

**Published:** 2014-11-02

**Authors:** Sébastien Gallien, Isabelle Charreau, Julien Fonsart, Samia Mourah, Issa Kalidi, Pierre Delobel, Bruno Marchou, Jean-Pierre Aboulker, Jean-Michel Molina

**Affiliations:** 1 Infectious Diseases and Tropical Medicine, Hôpital Saint-Louis, APHP, Université Paris Diderot, Paris, France; 2 INSERM, SC10, Villejuif, France; 3 Laboratory of Biochemistry, Hôpital Saint-Louis, APHP, Université Paris Diderot, Paris, France; 4 Pharmacology, Hôpital Saint-Louis, APHP, Université Paris Diderot, Paris, France; 5 Hematology Laboratory, Hôpital Saint-Louis, APHP, Université Paris Diderot, Paris, France; 6 Infectious Diseases, CHU Purpan, Toulouse, France

## Abstract

**Introduction:**

In the SMART trial, baseline plasma hsCRP, IL6 and D-dimer levels were strongly correlated to all-cause mortality. A case-control study has shown an increase of IL-6 and D-dimer levels after one month of antiretroviral therapy (ART) interruption, which was correlated to viral load. Restarting ART was associated to a decrease in D-dimer but not IL-6 or hsCRP levels. We assessed biomarkers levels up to 96 weeks in ART-experienced adults with plasma HIV RNA levels <400 c/mL randomized in the ANRS 106 WINDOW trial to intermittent ART (IT: six cycles of eight weeks of ART interruption followed by eight weeks of ART) versus continuous treatment (CT).

**Methods:**

Stored plasma for 160 participants (80 IT and 80 CT), matched by age, sex and CDC classification, were analyzed blinded for IL-6, sCD-14, hsCRP and D-dimer levels at baseline, week 8 (IT group only), week 16 and week 96. Lower levels of detection for IL-6, sCD14, hsCRP and D-Dimer were 1.5 pg/mL, 250 ng/mL, 0.03 µg/mL and 0.21 µg/mL, respectively. The primary objective was to compare changes in IL-6, hsCRP, sCD14 and D-dimer plasma levels from baseline to week 8, 16 and 96 in the IT and CT arms. Biomarkers levels were log10 transformed prior to analysis.

**Results:**

At baseline, patients were mostly men (86%), with a median age of 40 years, a CD4+ T-cell count of 768/mm^3^, have received a median of 4.7 years of ART and 85% had HIV RNA <50 c/mL. Proportion of patients with plasma HIV RNA levels<400 c/mL were 6% and 99%, 81% and 97%, 86% and 92% at weeks 8, 16 and 96 in the IT and CT arms, respectively. Plasma biomarkers levels are shown in the Table 1.

Compared to baseline, D-dimer levels significantly increased 8 weeks after ART interruption in the IT arm (+23% fold change, 95% CI +9% to +39%) but reverted to baseline levels at week 16 and remained unchanged at week 96. There was no significant change from baseline in the other biomarker levels in the IT arm. Similarly, no significant change from baseline in biomarker levels was seen in the CT arm up to 96 weeks.

**Conclusion:**

Coagulation and inflammatory biomarkers levels remained stable over 96 weeks in well-suppressed HIV-infected patient on ART. Following ART interruption there was a significant increase in D-dimer but not in inflammatory biomarkers levels. This increase was reversed upon reintroduction of ART. These data suggest that ART interruption increases coagulation rather than inflammatory biomarkers.

**Table 1:** Changes in biomarkers levels in participants from the intermittent (IT) vs continuous (CT) treatment arms through 96 weeks (*p=0.002, Wilcoxon test)

	IL-6(pg/mL)		sCD14(ng/mL)		hsCRP(#x00B5;g/mL)		D-dimer(µg/mL)	
	
	IT	CT	IT	CT	IT	CT	IT	CT
Crude baseline levels: Means (SD)	1.58 (0.35)	2.23 (3.42)	3196 (1172)	3310 (1467)	2.60 (3.51)	6.10 (15.86)	0.30 (0.20)	0.36 (0.34)
Percent changes (log10 transformed) Mean (95% CI)								
From BL to week 8 (off treatment)	−6 (−16; +5)	ND	+5 (−5; + 16)	ND	−9 (−32; + 22)	ND	+23* (+9; + 39)	ND
From BL to week 16 (on treatment)	−3 (−14; + 8)	+1 (−2; + 5)	+5 (−3; + 14)	+2 (−4; + 8)	−9 (−30; + 19)	+14 (−8; + 42)	+4 (−11; + 21)	+6 (−3; + 16)
From BL to week 96 (on treatment)	−1 (−9; + 8)	+8 (0; + 18)	0 (−8; + 10)	+6 (−1; + 14)	−11 (−30; + 13)	+19 (−6; + 52)	−3 (−14; + 10)	+10 (−4; + 27)

